# Swing-Arc Narrow-Gap Submerged Arc-Welding Process Assisted by Pre-Embedding Cold Wires

**DOI:** 10.3390/ma19081655

**Published:** 2026-04-21

**Authors:** Shubin Liu, Yupeng Cao, Hong Li, Jie Zhu, Changxin Zhou, Zhengyu Zhu, Jiayou Wang

**Affiliations:** 1 School of Materials Science and Engineering, Jiangsu University of Science and Technology, 666 Changhui Road, Zhenjiang 212100, China; shubin_l18@just.edu.cn (S.L.); 231110601207@stu.just.edu.cn (Y.C.); lihong@just.edu.cn (H.L.); zhujie-2010@just.edu.cn (J.Z.); wyyx3768@163.com (C.Z.); 2 School of Intelligent Welding Technology, Guangxi Technological College of Machinery and Electricity, 101 East Daxue Road, Nanning 530007, China

**Keywords:** narrow gap submerged arc welding, arc swing, cold wire, weld formation, joint microstructure

## Abstract

To solve the problems of poor weld formation, difficult slag removal, and inferior joint microstructure and hardness in conventional narrow-gap submerged arc welding (NG-SAW), a swing arc NG-SAW process assisted by pre-embedding cold wires was proposed. Synergistically optimizing the welding energy parameters and additional cold wires ensured sound weld formation and enhanced slag detachability, while the efficiency of multilayer welding was improved by reducing the number of weld layers by 33.3%. The slag adhesion mechanism is clarified as follows: a high welding heat input facilitates elemental diffusion at the weld–slag interface, leading to the formation of a continuous and thick interlayer composed of (Fe,Mn)O and MgO-Al_2_O_3_-CaO phases. This interlayer strengthens the chemical bonding between slag and weld, thereby impeding slag removal. Microstructure evolution analysis of the multilayer welded joint revealed that the variable-angle design increases the groove volume and, combined with the heat-absorbing effect of the additional wires, accelerates molten pool cooling, thereby refining grains in both the weld metal zone and reheat-affected zone. Meanwhile, the tempering exerted by the heat-affected zone (HAZ) of the subsequent weld layer on the previous layer is attenuated. This promotes the gradual transformation of hard-brittle lath martensite in the coarse-grained heat-affected zone (CGHAZ) of the bottom layer into tougher tempered martensite/bainite in the CGHAZ of the upper layers. As a result, the hardness uniformity within the HAZ, the critical weak region of the joint, was enhanced by 54%, enabling synchronous improvement in microstructural homogeneity, hardness distribution, and overall welding efficiency.

## 1. Introduction

With the rapid development of offshore equipment, pressure vessels, steel manufacturing, etc., heavy-walled equipment has been widely used, and the demand for efficient and high-performance arc welding technology is growing. Narrow-gap welding (NGW) is an efficient welding method for thick plates, featuring good joint performance and high welding efficiency. Accordingly, it has emerged as one of the research hotspots in the field [1,2]. It mainly includes narrow-gap gas metal arc welding (NG-GMAW) [3,4], narrow-gap submerged arc welding (NG-SAW) [5,6], and narrow-gap laser arc hybrid welding (NG-LAHW) [7]. However, owing to its characteristics of low-current operation, short-circuiting metal transfer, and controllable molten pool, gas metal arc welding (GMAW) [8,9] is more suitable for welding with fine-diameter wires (≤1.2 mm). Since laser arc hybrid welding is generally implemented in conjunction with GMAW, it not only inherits the above shortcomings but also is restricted by the trade-off between laser power and welding efficiency, making it unsuitable for thick-plate welding. In contrast, submerged arc welding (SAW) [10,11] is inherently capable of utilizing thick wires (e.g., 4.0 mm), which enables the high current input and large deposition rate required for thick-plate joining, thereby remarkably enhancing welding efficiency.

However, poor sidewall penetration is the main challenge of NGW. To solve this problem, several attractive rotating/weaving arc processes were developed, such as rotating arc [12,13], magnetically oscillating arc [14], and mechanically swinging arc [15,16]. Those methods utilized mechanical or magnetic force to rotate/weave the arc so as to regulate the heat and force distributions of the arc in a narrow-gap groove, and accordingly yield sufficient penetration into the groove sidewalls. Of those methods, arc swing has the advantages of good arc directivity and adjustable spatial position, which makes it easy to control the weld formation [17].

In parallel with process development, arc modeling has emerged as an indispensable approach for elucidating arc behavior under atmospheric-pressure conditions. Previous investigations [18,19] have demonstrated that fluid dynamics-based and non-equilibrium thermodynamic models can effectively capture key discharge characteristics, encompassing charged-particle transport, energy transfer mechanisms, near-electrode phenomena, and current-voltage responses. These studies further highlight that model predictions are highly sensitive to the assumed gas medium properties, boundary condition settings, electrode thermal states, and discharge geometric configurations. However, the majority of existing models have been developed specifically for simplified direct current discharges or gas-shielded arc systems. Their direct applicability to swing arc NG-SAW remains limited, primarily due to the unique complexities inherent to the latter process: these include a flux-covered narrow groove, slag detachability issues arising from slag–metal interfacial interactions, dynamic arc swing behaviors, and thick-wire high-deposition rate operations. Therefore, a comprehensive experimental characterization of the fundamental process characteristics of swing arc NG-SAW is a prerequisite to the development of a dedicated and physically sound mathematical model.

To further raise NGW efficiency, novel NG-GMAW processes with additional filling wire were reported [17,20,21]. Zhu et al. [21] have successfully realized a significant improvement in welding efficiency via a cold wire-assisted narrow-gap welding process. Notably, the above welding processes utilize the heat absorbed by the melting of additional cold wire to regulate the welding heat input, which can not only improve weld formation but also refine the weld structure and enhance its mechanical properties. However, the majority of existing cold wire-assisted technologies are primarily tailored for NG-GMAW, and few studies have attempted to apply cold wire assistance specifically to thick-wire NG-SAW. Given that the relatively high heat input of SAW tends to deteriorate the controllability of weld formation, applying the advantages of cold wire assistance in regulating weld formation to NG-SAW is anticipated to fill this research void.

In addition, the reduction in groove gap and the increase in welding heat input tend to cause severe accumulation and adhesion of slag within the narrow groove space, making it difficult for the slag to detach naturally. Currently, two main strategies are adopted in existing technologies to address the aforementioned slag removal issue. First, an NG-SAW process employing two or more passes per layer is utilized [22], which leverages the free shrinkage of single-pass welds in the groove to mitigate slag adhesion and thereby improve slag detachability. Nevertheless, this technique requires at least two weld passes per layer, which considerably increases the number of welding procedures and significantly undermines the inherent high-efficiency advantage of NG-SAW. Second, specialized slag removal equipment, such as slag crushers and wire brushes, is commonly used to forcibly remove slag from narrow grooves [23]. This approach relies on an additional slag removal apparatus and dedicated processes, which not only increase production costs but are also inefficient. Collectively, these methods fail to fundamentally resolve the inherent trade-off between difficult slag removal and enhanced welding efficiency in NG-SAW, and therefore cannot meet the industrial requirements for high-efficiency and high-quality welding.

Accordingly, to address the aforementioned core challenges of NG-SAW, including insufficient sidewall penetration, difficult slag removal, and low efficiency, this study presented a novel single-pass-per-layer NG-SAW process featuring a thick-wire swing arc assisted by pre-embedding cold wires. The influences of welding heat input and additional cold wires on weld formation and slag detachability were systematically investigated. A predictive model for slag removal was established to optimize the welding process parameters. Furthermore, the mechanism of slag adhesion in the NG-SAW process was elucidated. Meanwhile, the multilayer welding efficiencies with and without cold wire assistance were evaluated. Finally, the microstructural evolution and hardness performance of multilayer welded joints were examined. This work provides a theoretical and technical foundation for the industrial application of high-efficiency NG-SAW in the manufacturing of heavy-walled equipment.

## 2. Materials and Methods

### 2.1. Experimental Setup

**Figure 1:**
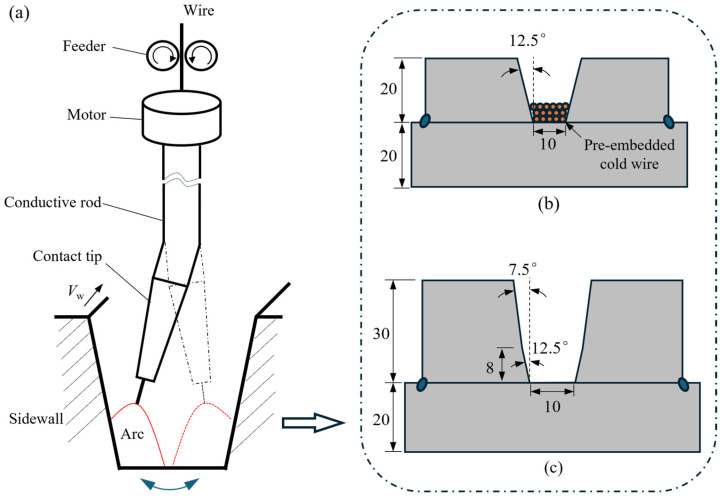
Schematic diagram of the swing arc NG-SAW process: (**a**) system configuration, and the cross-sectional dimensions of test pieces for (**b**) single-layer welding and (**c**) multilayer welding.

Figure 1 illustrates the schematic diagram of the swing arc NG-SAW process. A welding supply (MZ-1250, Aotai, Jinan, China), which has a descending output characteristic and matches a wire feeder with a variable rate, was used for submerged arc welding. The welding torch is independently designed, which mainly consists of a hollow-axis motor, a bending conductive rod, and a contact tip. The welding wire is fed to pass successively through the motor, bending rod, and contact tip, and an arc finally burns between the wire and workpiece. The motor of hollow axis periodically turns the upwardly bending rod at an angular velocity of *ω*, and then drives the arc to swing left and right within the groove, as shown in Figure 1a. The arc movement is constrained by the parameters of swing angle *α*, swing frequency *f*, and staying time *t_s_* at the near sidewalls (where a reserved gap (*g*) is defined as the minimum distance from the arc to the sidewall) [21].

### 2.2. Materials and Welding Process

A thick welding wire (CHW-S3, ATLANTIC, Zigong, China) with a diameter of 4.0 mm was used for the NG-SAW process, and EH36 steel was used as the base metal. The chemical compositions of the base metal and welding wire are listed in Table 1. In addition, the pre-embedded cold wire is a straight rod, shaped with a diameter of 2.0 mm, which has a chemical composition that is the same as the main wire. The flux used is a code of CHF101 (ATLANTIC, China), which matches the main welding wire. The workpieces were assembled using three steel plates and featured a narrow V-shaped groove, whose cross-sectional geometry is illustrated in Figure 1b,c. Note that, to meet the slag removal requirements and further improve welding efficiency, a groove with variable angles was employed for multilayer welding, as shown in Figure 1c.

Statistical analysis of numerous welding test results showed that, in the NG-SAW process using the grooves shown in Figure 1b,c, the main factors affecting slag removal and weld formation are welding heat input and the additional cold wire. Accordingly, a series of narrow-gap welding experiments was conducted, as presented in Table 2, to investigate the effects of welding heat input and additional cold wires on weld formation and slag removal. The arc swing parameters were determined based on empirical data to ensure sufficient sidewall penetration. For single-layer welding, up to three layers of cold wires can be embedded at the bottom of the groove; more than three layers may result in underfusion at the bottom. For multilayer welding, three layers of cold wire were pre-embedded before each welding pass. Furthermore, due to the use of a variable groove angle, the total cold wire for each welding pass varies, as shown in Table 3. During the welding process, the preheating temperature of the test piece before welding and the interlayer temperature were controlled at ~150 °C. In addition, to ensure the stability of the welding process, three replicate specimens were welded for each set of welding parameters, and the stability of the welding process was evaluated through the examination of weld appearance and cross-sectional formation.

It should be emphasized that the welding current (*I_a_*), arc voltage (*U_a_*), and welding speed (*V_w_*) collectively determine the welding heat input *E*, which can be calculated by:

(1) $$E=\frac{I_{a}\cdot U_{a}}{V_{w}}$$

Since all three parameters govern the heat energy absorbed per unit length of the weld metal via the same physical mechanism, their influences on weld formation and slag detachability exhibit consistent evolutionary trends. For simplicity and clarity of analysis, the welding current (Table 2) was therefore selected as the representative parameter to demonstrate the welding heat input effect in the following discussion.

### 2.3. Microstructure Characterization

**Figure 2:**
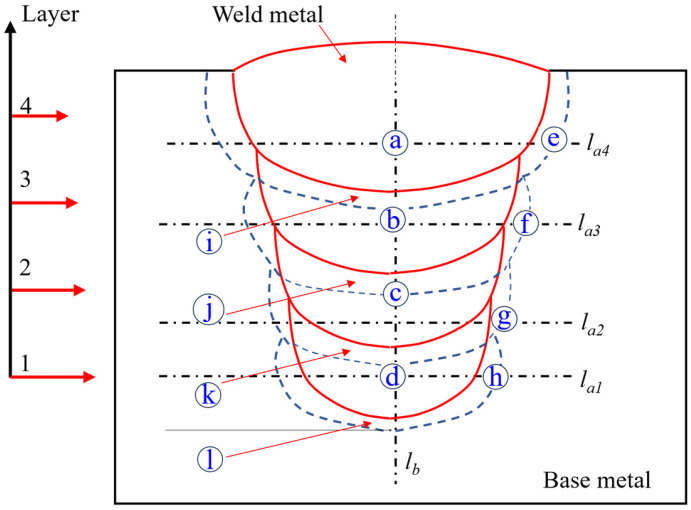
Illustration of the regions for microstructure observation and hardness testing in the multilayer welded joint.

The microstructural analysis includes SEM observation and metallographic observation of the cross-sectional welding joints. During the pretreatment of the two types of specimens, the oxide layer was removed by mechanically grinding with #240~#2000 emery papers and then polishing with 3.5 μm WC suspension until no visible scratches remained. For SEM observation of the joints, the micromorphology and elemental mapping analysis were performed with a Mira field emission SEM (Hitachi, SU8600, Tokyo, Japan) equipped with an EDS system (Oxford, Ultim max 40, High Wycombe, England) at an acceleration voltage of 8 kV. For metallographic observation of the joints, the specimens were etched with a 4% nitric acid–ethanol solution for 9 s. Macro-images of the joints were then taken using a macro digital camera, and the microstructure characteristics of the joints were observed using an inverted optical microscope (Zeiss, Axio Observer.3m, Oberkochen, Germany). The regions for metallographic observation are shown in Figure 2, where regions a–d are located in the weld metal zone, regions e–h are located in the sidewall heat-affected zone (HAZ), region l is located in the bottom HAZ, and regions i–k are located in the reheat-affected zone (RHAZ).

### 2.4. Hardness Testing

An automatic hardness tester (KB, 30S FA BASIC, Hochdorf-Assenheim, Germany) was employed to measure the Vickers hardness of the specimens at a load of 0.5 kgf for 10 s. The hardness testing points were taken along dashed axes *l_a_*_1_–*l_a_*_4_ and *l_b_*, where there was a sampling interval of 0.5 mm in the weld metal region, 0.1 mm in RHAZ and HAZ, and 1 mm in the base metal region, as shown in Figure 2. The hardness uniformity was quantitatively assessed by the coefficient of variation (CV), defined as the ratio of the standard deviation (SD) to the average hardness value; a lower CV value signifies a more homogeneous hardness distribution throughout the joint. The improvement in hardness uniformity (Δ*CV*) was further evaluated by calculating the relative reduction in CV between the cold wire-assisted process and the conventional process without cold wire addition:

(2) $$∆CV=\frac{{CV}_{0}-{CV}_{cw}}{{CV}_{0}}\times 100\%$$

where *CV*_0_ denotes the coefficient of variation in the conventional process without cold wire addition, and *CV_cw_* represents that of the cold wire-assisted process.

## 3. Results and Discussion

### 3.1. Narrow-Gap Welding Characteristics

#### 3.1.1. Single-Layer Weld Formation and Slag Detachability

**Figure 3:**
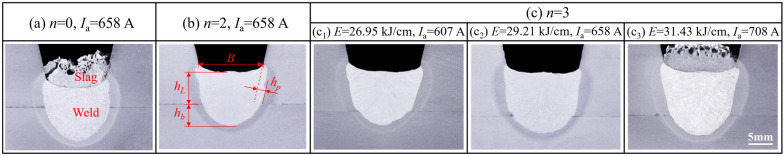
Effect of pre-embedded cold wire layer (*n*) and welding heat input (*E*) on narrow-gap weld formation and slag detachability: (**a**–**c**) the effect of *n*, and (**c_1_**–**c_3_**) the effect of *E*.

**Figure 4:**
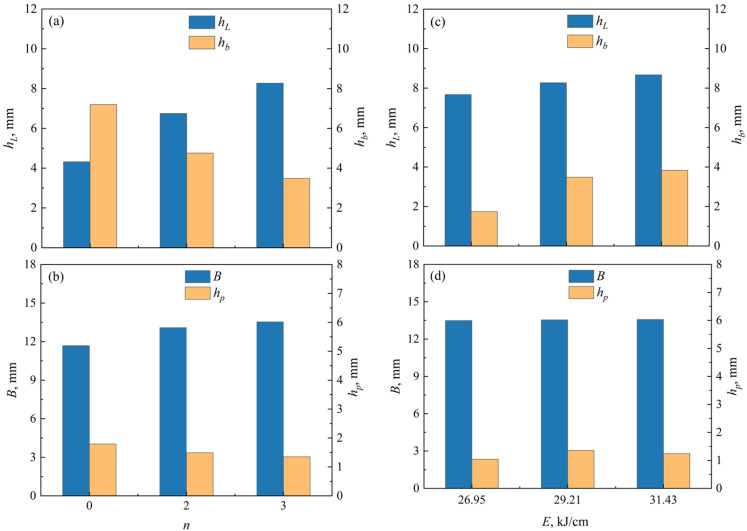
Effect of pre-embedded cold wire layer (*n*) and welding heat input (*E*) on narrow-gap weld shape parameters: (**a**,**c**) weld cladding thickness (*h_L_*) and bottom penetration (*h_b_*), and (**b**,**d**) weld width (*B*) and sidewall penetration (*h_p_*).

Figure 3 shows the effect of pre-embedded cold wire layer and welding heat input on narrow-gap weld formation and slag detachability. Figure 3b illustrates the key weld shape parameters, where *h_L_* is the weld cladding thickness, *h_b_* and *h_p_* denote the bottom penetration and sidewall penetration respectively, and *B* represents the weld width. Figure 4 presents the data analysis of the weld shape parameters, where *h_p_* is the average of the left and right sidewall penetrations.

In the absence of cold wires (*n* = 0), severe slag adhesion appears on the bead, as shown in Figure 3a, even though relatively sufficient sidewall penetration was yielded (see Figure 4b). With the addition of cold wires, slag detachability is notably improved owing to the heat-absorbing effect introduced by the cold wires. In addition, as the number of cold wire layers increases from 0 to 3 (Figure 3(b,c_2_)), the effective heat-absorbing zone expands accordingly. Simultaneously, the increased filler metal volume prevents the arc from directly heating the groove bottom, leading to a gradual reduction in bottom penetration (*h_b_*) and sidewall penetration (*h_p_*). In contrast, the weld cladding thickness (*h_L_*) and weld width (*B*) increase with rising filler metal volume (see Figure 4a,b). It is worth noting that the bottom and sidewall penetrations (*h_b_* and *h_p_*) still remain at a relatively high level despite the slight decrease.

At a fixed cold wire layer number of *n* = 3, increasing the welding heat input from 26.95 kJ/cm to 31.43 kJ/cm (see Figure 3(c_1_–c_3_)) enhances the arc energy imposed on the groove region, resulting in increased bottom penetration (*h_b_*) and sidewall penetration (*h_p_*). However, the weld cladding metal thickness (*h_L_*) and weld width (*B*) show no obvious variation with the increasing welding heat input, because the net deposited metal volume per unit weld length remains nearly constant (see Figure 4c,d). Nevertheless, when the welding heat input exceeds 29.21 kJ/cm, the slag bonds strongly to the weld surface, as shown in Figure 3(c_3_). It follows that, under the premise of ensuring satisfactory slag detachability, the maximum allowable welding heat input increases with pre-embedded cold wire layers. Evidently, for the customized narrow-gap dimensions employed in the present study, the effective welding heat input should not exceed 29.21 kJ/cm; exceeding this threshold would induce severe slag adhesion and thereby compromise the integrity and quality of the welded joint.

**Figure 5:**
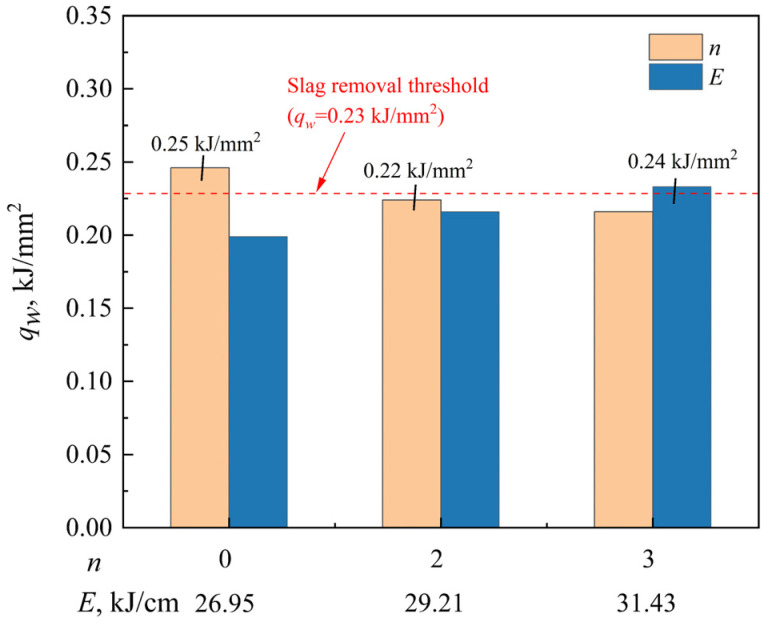
Effect of pre-embedded cold wire layer (*n*) and welding heat input (*E*) on arc energy density on the weld surface (*q_w_*).

Excellent slag detachability is essential for the stable and high-quality operation of the NG-SAW process. Insufficient slag removal tends to induce welding defects such as slag inclusions, which further degrade the mechanical performance of the welded joint. To quantitatively evaluate and predict slag detachability, a variant of arc energy density on the weld surface, *q_w_*, was proposed, with its expression defined as:

(3) $$q_{w}=\frac{U_{a}\cdot I_{a}}{V_{w}\cdot B}$$

where *U_a_* is the arc voltage, *I_a_* is the welding current, *V_w_* represents the welding speed, and *B* denotes the weld width (see Figure 3b). Calculated *q_w_* values under different welding conditions presented in Figure 3 are plotted in Figure 5. The results clearly reveal that slag removal deteriorates significantly when *q_w_* exceeds 0.23 kJ/mm^2^, which agrees well with the severe slag adhesion phenomenon observed in Figure 3(a,c_3_). Importantly, the threshold of *q_w_* = 0.23 kJ/mm^2^ is established specifically for the narrow-gap geometry and welding material system adopted in the present study. For different engineering conditions, such as altered groove angles, base metal grades, or flux systems, the threshold value of *q_w_* should be recalibrated to ensure reliable prediction of slag detachability.

It follows that the proposed arc energy density parameter *q_w_* can serve as a reliable quantitative indicator for evaluating slag detachability in NG-SAW, which provides a valuable theoretical reference for optimizing welding parameters to achieve favorable deslagging behavior and high-quality weld formation.

#### 3.1.2. Slag Adhesion Mechanism

During the NG-SAW process, difficult slag removal has become a serious challenge that affects joint formation and welding efficiency. In this study, the interfacial reaction between the slag and the weld was analyzed from a microscopic perspective to reveal the slag adhesion mechanism, which is expected to provide theoretical guidance for optimizing the NG-SAW process.

**Figure 6:**
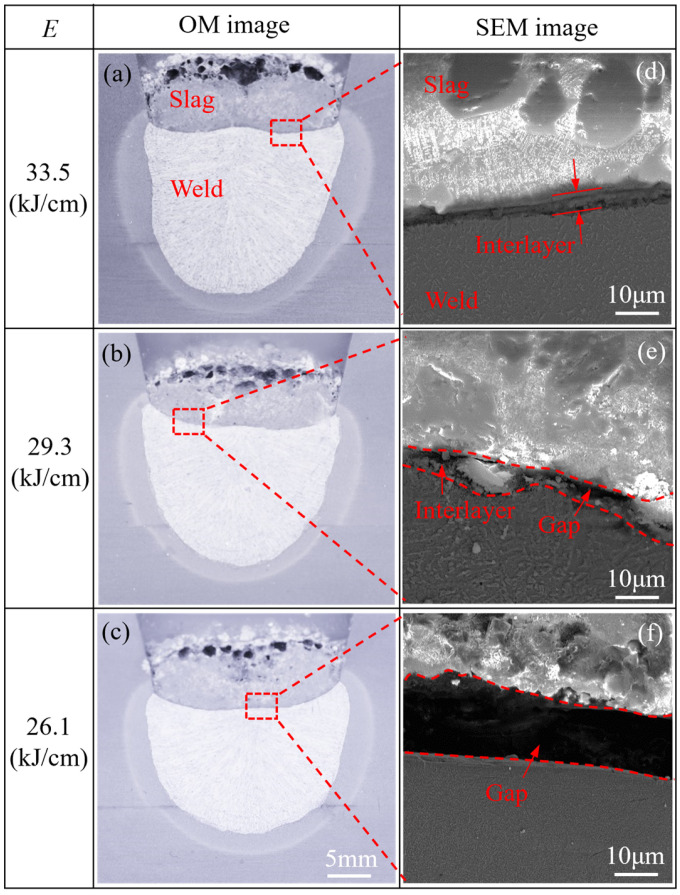
Effect of welding heat input (*E*) on slag removal: (**a**–**c**) are OM images of the cross-section of the welds, and (**d**–**f**) are SEM images of observation areas corresponding to (**a**–**c**).

Figure 6 shows the adhesion characteristics between the weld and the slag under different welding heat inputs (*E*). Among them, Figure 6a–c presents the optical microscopy (OM) images of the cross-section of the welded joints. From the macrographs, the welds produced under all three heat input conditions exhibit obvious bonding with the slag, making it difficult to judge the ease of slag removal from the macromorphology. In contrast, the scanning electron microscopy (SEM) images of the weld–slag interface clearly show the bonding state under all three heat inputs, as illustrated in Figure 6d–f. From the micrographs, the upper part is the slag, the lower part is the weld, and a reaction interlayer or gap is present in the middle. Deslagging becomes difficult at *E* = 33.5 kJ/cm, which can be attributed to the formation of a reaction interlayer between the slag and the weld (see Figure 6d). When *E* decreases to 29.3 kJ/cm, the difficulty of deslagging is moderately reduced, with only a small amount of residue adhering to the weld. This may be due to the formation of an incomplete, discontinuous interlayer, as shown in Figure 6e. A wide gap is formed between the slag and the weld at *E* = 26.1 kJ/cm (see Figure 6f). Consequently, the slag is completely separated from the weld, enabling easy slag removal. Accordingly, it can be inferred that the formation of a continuous reaction interlayer is the primary factor responsible for difficult deslagging.

**Figure 7:**
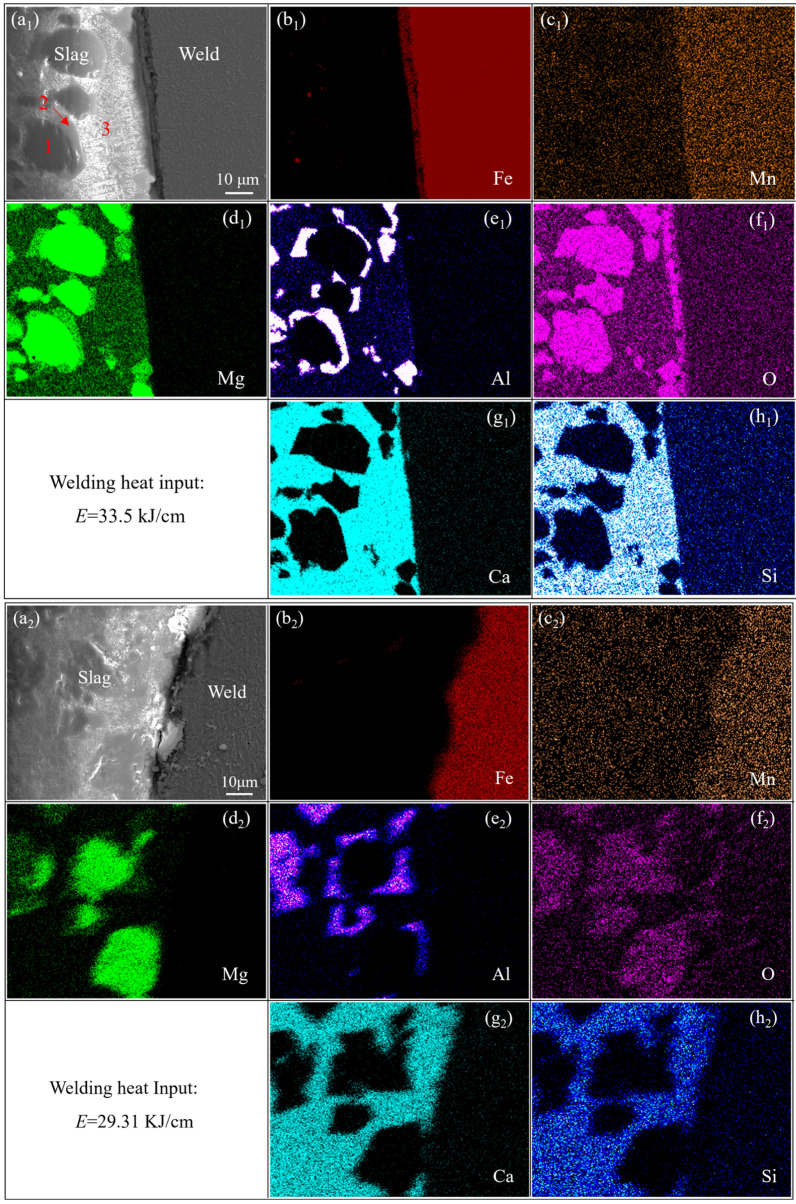
SEM images and element distributions of the junction region between the weld and slag under welding heat inputs of 33.5 kJ/cm and 29.31 kJ/cm, respectively: (**a_1_**,**a_2_**) SEM images, and (**b_1_**–**h_1_**) and (**b_2_**–**h_2_**) element distributions.

**Figure 8:**
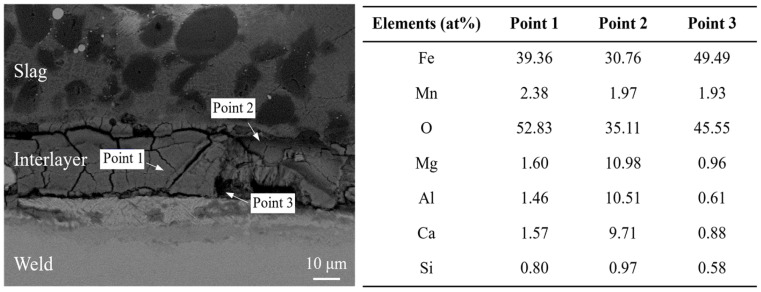
Quantitative elemental analysis of the interlayer.

To further understand the formation mechanism of the interlayer, micro-region elemental analysis of the weld–slag bonding interface was performed, as shown in Figure 7. From the elemental distribution maps (see Figure 7(d_1_–h_1_,d_2_–h_2_)), it can be concluded that the slag is mainly composed of magnesite (MgO, Phase 1), aluminum–magnesium spinel (MgO-Al_2_O_3_, Phase 2) encapsulated on the surface of magnesite, and a matrix of rock phase compounds (SiO_2_-CaO-MgO-Al_2_O_3_, Phase 3). In addition, based on the elemental distribution across the bonding interface between the weld and the slag, the bonding interface was found to contain elements such as Fe, Mn, Mg, Al, Ca, and O (see Figure 7(b_1_–h_1_)). Quantitative elemental analysis was further performed on the interlayer region, with the corresponding results presented in Figure 8. It is revealed that the interlayer is predominantly composed of Fe and O, while Mn is homogeneously distributed throughout the interlayer. Meanwhile, Mg, Ca, and Al are mainly enriched on the slag-proximal side. Based on these findings, it is inferred that an interlayer of rock phase compounds, dominated by (Fe,Mn)O with a small amount of MgO-Al_2_O_3_-CaO [24,25], was formed. It should be noted that this interfacial reaction mechanism is primarily deduced from the SEM/EDS observations of elemental distribution. To gain deeper insights into the phase composition and crystal structure of these interlayer phases, further investigations, such as XRD analysis of the interlayer, are indispensable. These phases facilitate the formation of a gelatinous slag layer (i.e., the reaction interlayer) by tightly bonding to the weld surface.

In contrast, when the welding heat input is 29.31 kJ/cm, almost no Fe diffuses from the weld into the slag, and simultaneously, the diffusion amount of Al, Ca, and Si from the slag into the weld decreases synchronously (see Figure 7(b_2_–h_2_)). Accordingly, no continuous reaction interlayer is formed (or only a discontinuous, incomplete interlayer exists) between the weld and the slag.

**Figure 9:**
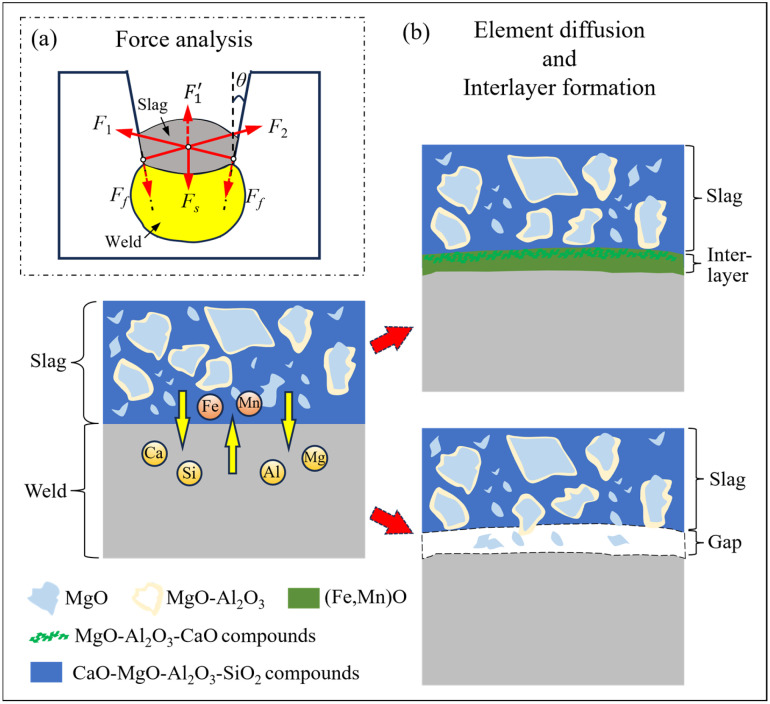
Schematic of the slag adhesion mechanism: (**a**) force analysis for the slag and (**b**) element diffusion and interlayer formation at the weld–slag interface.

Actually, the weld metal undergoes transverse shrinkage during cooling and solidification. The overlying molten slag layer remains partially molten or semi-solid at elevated temperatures during the early stage of weld shrinkage. Full solidification of the slag occurs only at relatively low temperatures, after weld shrinkage has largely been completed. Compared with the molten weld metal, the slag has a smaller linear expansion coefficient, resulting in smaller shrinkage during the cooling process. Since the slag is not fully solidified when the weld commences shrinkage, its elastic resistance is negligible, and yet it imposes continuous lateral constraint at the groove interface. Accordingly, when the weld metal shrinks transversely along the groove, it is constrained by the rigid groove, causing the sidewall to exert a compressive force (*F*_1_ = *F*_2_) on the slag. This force is perpendicular to the sidewall and points towards the center of the slag, as shown in Figure 9a. The compressive force, which is closely related to the transverse shrinkage of the weld metal and the mechanical properties of the materials, can be indirectly estimated via the following series of expressions, where empirical coefficients and parameter definitions are clearly specified:

(4) $$∆B=\frac{0.18\cdot A_{w}}{\delta }$$

(5) $$\varepsilon =\frac{∆B}{L}$$

(6) $$F_{1}=F_{2}=E\cdot A_{w}\cdot \varepsilon$$

(7) $$F_{1}^{\prime }=F_{1}\cdot sin\theta$$

where Δ*B* denotes the shrinkage of the weld metal along the transverse direction of the groove (unit: mm); *A_w_* represents the cross-sectional area of the weld (unit: mm^2^), which was measured via image processing of the weld cross-sectional metallographic samples; *δ* is the thickness of the sidewall (*δ* = 20 mm, as shown in Figure 1b; unit: mm); *L* is the root clearance of the groove (*L* = 10 mm, as shown in Figure 1b; unit: mm); *E* is the elastic modulus of weld metal (unit: GPa), which is measured by uniaxial tensile tests of the weld metal samples, with a value of 205 GPa, which is consistent with the low-carbon steel weld metal used in this study; and *ε* denotes the shrinkage strain of the weld metal in the transverse direction (dimensionless). We assumed that the transverse shrinkage of the weld metal is uniform along the weld length, and the shrinkage strain (*ε*) is evenly distributed over the weld cross-section. This assumption is consistent with experimental observations that uniform weld formation is achieved under the optimized welding parameters employed in this study. The empirical coefficient 0.18 in Equation (4) is derived from the experimental data of this study and verified by relevant literature [26]. Specifically, welding tests were conducted with different weld cross-sectional areas (*A_w_*) under the same experimental conditions (fixed *δ* = 20 mm), and the corresponding transverse shrinkage (Δ*B*) was measured. Linear fitting of the experimental data (Δ*B* vs. *A_w_*/*δ*) yielded a fitting line slope of 0.18.

In addition, because the welding wire, cold wire, and the workpiece dimensions employed were identical, parameters *E*, *δ*, and *L* in expressions (4)–(6) are considered constant. Consequently, the compressive force *F*_1_ is proportional to *A_w_*. A higher welding heat input leads to more base material melting, resulting in a larger cross-sectional area of the weld (*A_w_*). Accordingly, the compressive force *F*_1_ is positively correlated with the introduced welding heat input. Furthermore, under the effect of *F*_1_ and *F*_2_, the slag experiences a frictional force (*F_f_*) downwards along the sidewall, which is expressed as:

(8) $$F_{f}=\mu \cdot F_{1}$$

where *μ* is the coefficient of friction between the slag and the groove sidewall (dimensionless). This frictional force is generated when the slag is still semi-solid and adheres to the bevel sidewall, and is completely locked in after the slag solidifies. Based on the typical friction coefficient range (0.1–0.3) between slag and steel at 25 °C [27], *μ* was set to 0.2 in this study, which is consistent with the material properties.

On the other hand, the interfacial tension of the molten slag and the interfacial tension at the slag–molten weld metal interface synergistically promote the wetting of the molten weld metal by the slag, enabling tight contact at the molecular level and generating physical adsorption forces (i.e., van der Waals forces). Additionally, a series of chemical metallurgical reactions occurs at the interface between the weld metal and slag, forming a transition layer that constitutes a chemical metallurgical bond. Ultimately, the slag and weld metal form an adhesive bonding force (*F_s_*) through the combined effect of “physical adsorption + chemical bonding”. It should be noted that the magnitude of *F_s_* is the result of the coupled effects of multiple factors, including the thickness of the interfacial transition layer, the chemical composition and crystal structure of the transition layer, the interfacial energy between the slag and the metal, and the stress compatibility during the cooling process. Therefore, there is currently no general quantitative formula for calculating the bonding force *F_s_* between slag and weld metal. The value of *F_s_* can be obtained under specific working conditions through subsequent experimental tests (such as slag peeling tests and interface tensile tests), which will be conducted in our follow-up research to further quantify the mechanical model.

Moreover, the physical adsorption force, arising from intermolecular van der Waals forces at the slag–weld metal interface, is weak and short-ranged. In contrast, chemical metallurgical bonding involves the formation of a transition layer with chemical bonding characteristics through chemical reactions at the interface. Obviously, when an interlayer is formed, the metallurgical bonding force dominates the overall bonding strength. Accordingly, within a reasonable range of welding heat input, a higher heat input contributes to a thicker interfacial transition layer, thereby enhancing the adhesive bonding force *F_s_*. However, excessively high heat input may cause coarsening of the transition layer structure or interfacial oxidation, which would counteract the enhancement of the bonding force.

So, the resultant force (*F_R_*) acting on the slag can be expressed as:

(9) $$F_{R}=F_{1}^{\prime }-(F_{s}+F_{f}\cdot cos\theta )$$

Therefore, the slag detachability is closely related to the resultant force *F_R_*. It should be noted that, for a multilayer welded joint, the mechanical stress analysis of the weld metal and slag for each welding layer is consistent with the above description. However, since multilayer welding uses a double V-groove, starting from the 2nd layer, the variable *L* in expression (5) represents the transverse width of the weld surface, i.e., *L_N_* (*N* = 1~3) denotes the transverse width of the *N*-th weld layer surface. This can be calculated based on the groove angle (*θ*) and the current weld cladding thickness (*h_NL_*, *N* = 1~3), i.e., *L_N_* = 10 + 2*h_NL_*·tan*θ*.

Figure 9b illustrates the interfacial reaction mechanism between the weld metal and slag during the NG-SAW process. When a high welding heat input is applied, it prolongs the high-temperature dwell time of the weld metal prior to solidification, which enhances the elemental transfer between the slag and the weld (see Figure 7(b_1_–h_1_)). In the high-temperature arc zone, which is mainly concentrated in the middle of the weld, minor amounts of SiO_2_, Al_2_O_3_, and MgO in the flux are ionized in the arc region, releasing ionized oxygen species. Some of this ionized oxygen combines with the elements of the high-temperature vaporized weld metal to form metal oxides (i.e., (Fe,Mn)O) [24]. These oxides enter the molten pool under the action of the arc and subsequently float to the surface of the weld metal as the molten pool solidifies.

In addition, the Mg, Al, and Ca elements generated by arc ionization diffuse into the molten weld metal. On the side near the slag interface, these elements dissolved in the molten weld metal are oxidized by SiO_2_ to form the MgO-Al_2_O_3_-CaO phase [25]. This formed phase, together with (Fe,Mn)O, constitutes the reaction interlayer between the weld metal and the slag (see Figure 9b). Since the density of these metal oxides is lower than that of the weld deposit metal, they float to the interface between the liquid metal surface and the slag, forming an intermediate layer with a coherent structure with the deposited metal during the solidification process. On the other hand, when the welding heat input decreases, the above elemental diffusion process is weakened, resulting in a thinner reaction interlayer with a weaker bonding force (*F_s_*).

In summary, based on the formation characteristics of the interfacial transition layer, the resultant force (*F_R_*) acting on the slag can be classified into three distinct cases, which correspond to different welding heat input conditions and subsequent slag detachability behaviors (detailed in Table 4):

i. Under high welding heat input conditions (*E* = 33.5 kJ/cm), a relatively thick and continuous interfacial transition layer is formed at the slag–weld interface (see Figure 6d). Under these circumstances, the adhesive bonding force (*F_s_*) dominates the force balance system, and the sum of *F_s_* and the downward component of the frictional force (*F_f_*·cos *θ*) is significantly larger than the upward component of the compressive force (*F*_1_′), corresponding to *F_R_* << 0. As a result, the slag forms an extremely tight adhesion with the weld metal and groove sidewall, making it extremely difficult to peel off.
ii. Under medium welding heat input conditions (*E* = 29.3 kJ/cm), the interfacial transition layer becomes thinner or discontinuous (see Figure 6e), leading to a noticeable reduction in the adhesive bonding force (*F_s_*). Nevertheless, *F_s_* still exceeds *F*_1_′, corresponding to *F_R_* < 0. In this scenario, the slag remains adhered to the weld surface and groove, and external force is required to peel it off.
iii. Under low welding heat input conditions (*E* = 26.1 kJ/cm), no interfacial transition layer is formed (see Figure 6f), and the adhesive bonding force (*F_s_*) is nearly zero. At this point, *F*_1_′ is essentially balanced with *F_f_*·cos *θ*, corresponding to *F_R_* ≈ 0. Under such conditions, even slight vibration can easily peel the slag off the weld surface.

#### 3.1.3. Multilayer Weld Formation and Welding Efficiency

**Figure 10:**
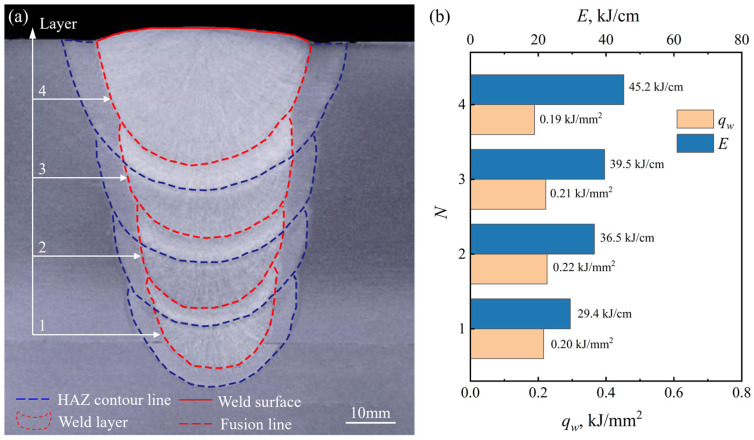
Cross-sectional weld formation of narrow-gap multilayer welded joint: (**a**) macrograph, and (**b**) weld surface arc energy density (*q_w_*) and welding heat input (*E*) for each weld layer (*N* represents the *N*-th weld layer).

Figure 10 illustrates the cross-sectional weld formation of the narrow-gap multilayer welded joint. As shown in Figure 10a, the sidewall and bottom penetrations are sufficient while the filling between weld layers is excellent, indicating that the proposed process is feasible. The *q_w_* for each weld layer was calculated according to the mathematical model for predicting slag detachability proposed in Section 3.1.1, as shown in Figure 10b. A maximum *q_w_* of 0.22 kJ/mm^2^ was obtained, which is below the threshold of 0.23 kJ/mm^2^, ensuring good slag detachability in the narrow-gap multilayer welding process. Note that the value of *q_w_* does not gradually increase with the increase in welding heat input, as shown in Figure 10b. This can be attributed to the employment of a variable-angle V-groove (see Figure 1c), which leads the weld width *B* to increase layer by layer, thereby suppressing the increase in *q_w_*.

In addition, to ensure proper deslagging, six weld layers are required to fill the groove with a thickness of 30 mm for the conventional narrow-gap welding process. By comparison, only four layers are needed to fill the same groove with additional cold wires. Accordingly, the total weld layer number was reduced by 33.3%. On the other hand, considering welding time (excluding pre-welding preparation and post-welding treatment times in the analysis), the significant reduction in weld layers contributes to a substantial shortening of the total welding duration. Specifically, the SAW process with pre-embedded wires has a welding time of 240 s, while the conventional SAW process without pre-embedded wires requires 641 s. Correspondingly, the integration of pre-embedded wires enhances the welding efficiency by 62.6%. Collectively, these improvements comprehensively promote the efficiency of the multilayer welding process, beyond the sole advantage of reducing weld layers.

### 3.2. Microstructure Evolution of Multilayer Welded Joint

#### 3.2.1. Weld Metal Zone

**Figure 11:**
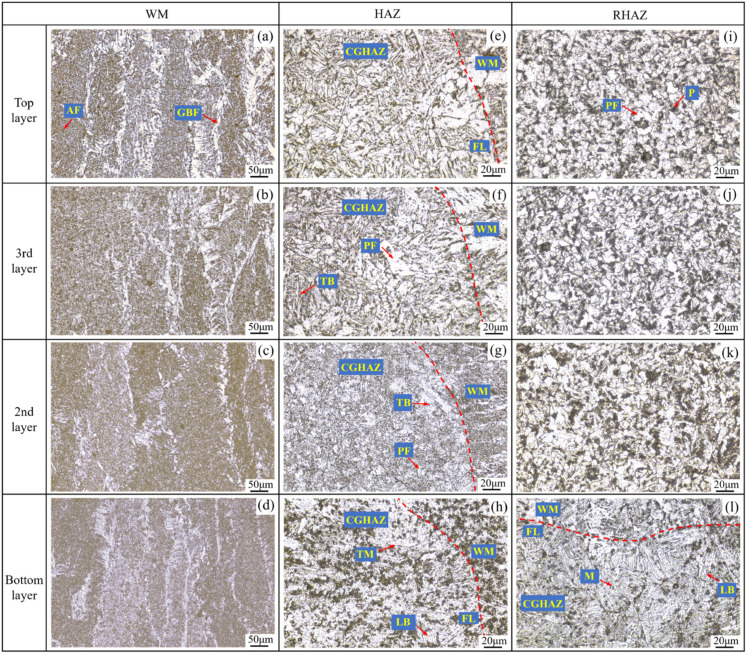
Microstructure of multilayer welded joint: (**a**–**d**) weld metal (WM) zone, (**e**–**h**) coarse-grained heat-affected zone (CGHAZ), (**i**–**k**) reheated-affected zone (RHAZ), and (**l**) CGHAZ in base metal.

Figure 11 shows the microstructures of the multilayer welded joint, where the microstructure observation regions correspond to those illustrated in Figure 2. The weld metal (WM) zones, presented in Figure 11a–d, are mainly composed of acicular ferrite (AF) and grain boundary ferrite (GBF), which develop via diffusion-controlled ferrite transformation during weld solidification owing to the low carbon level (C < 0.08% in Table 1) in the welding wire [28]. Compared with the bottom layer, the austenite grain size in the WM zone increases in the other three weld layers (see Figure 11b–d). Such microstructural evolution behavior is primarily governed by the thermal cycling characteristics during multilayer welding and the solidification dynamics of the molten pool. Specifically, the sequentially increasing heat input (see Figure 10b) and interlayer thermal accumulation significantly elevate the peak temperature and prolong the high-temperature dwell time of the welding thermal cycle, thereby promoting austenitic grain coarsening [29]. However, compared with the 2nd weld layer, the austenite grain sizes in the 3rd and top layers are slightly refined (see Figure 11a–c). This can be attributed to the beveled workpiece groove, which enlarges the molten pool cross-sectional area as the weld layer number increases. Combined with the heat-absorbing effect of cold wire melting, this accelerates the molten pool cooling rate and thus suppresses further growth of austenite grains to a certain extent. It should be noted that quantitative thermal cycle data were not obtained in the present work. Therefore, the above analysis of thermal effects on grain size evolution remains semi-quantitative and relies on process parameters and microstructural evidence. A more rigorous correlation between thermal cycles, cooling rates, and austenite grain growth kinetics would require dedicated thermal simulation or in situ temperature measurements, which is recommended as a direction for future investigation.

#### 3.2.2. Heat-Affected Zone

The coarse-grained heat-affected zone (CGHAZ) adjacent to the fusion line (FL) is generally recognized as the weakest region within the heat-affected zone (HAZ) of welded joints, as its microstructural characteristics exert a decisive influence on the overall mechanical properties and service reliability of the welded joints. The microstructural characterization results of the CGHAZ are presented in Figure 11e–h,l. For the bottom weld layer, due to the low initial temperature of the base metal and minimal welding heat input, the molten pool cools at a relatively fast rate. Under such rapid cooling conditions, the austenite transformation in the CGHAZ is insufficient. Consequently, the metastable austenite undergoes a shear-dominated transformation preferentially, giving rise to a hardened microstructure dominated by lath martensite (LM) and fine lath bainite (LB), as illustrated in Figure 11l.

During the welding process of the 2nd layer, when the arc swings near the sidewalls, the arc heat exerts a tempering effect on the sidewall CGHAZ of the bottom layer, causing the original martensite structure in this zone to soften and transform into tempered martensite (TM) with a slightly lower hardness (see Figure 11h). Starting from the 2nd weld layer, the increasing heat input reduces the cooling rate of the molten pool, leading to the sidewall CGHAZ of each subsequent layer (i.e., 2nd layer to top layer) being dominated by bainite and a small amount of polygonal ferrite (PF), whose toughness is superior to that of GBF. This microstructure evolution trend is consistent with the research results of Sun et al. [30].

Furthermore, under the tempering effect applied by the subsequent weld layer, the microstructure in the current layer transforms into more stable, lower-hardness tempered bainite (TB), and the PF grains coarsen (see Figure 11f,g). In addition, although the top layer is not affected by tempering from subsequent weld layers, it has the highest welding heat input, resulting in a microstructure in its sidewall CGHAZ (see Figure 11e) that is similar to that of the side CGHAZ in the 3rd layer.

#### 3.2.3. Reheat-Affected Zone

Figure 11i–k shows the microstructure of the reheat-affected zone (RHAZ) between the weld layers. The structural characteristics of the RHAZ differ significantly from those of the WM zone (see Figure 11a–d), mainly consisting of fine PF and pearlite (P). Although the multilayer welding process employs a layer-by-layer increasing welding heat input, the increased groove angle in the 3rd and top layers enlarges the cross-sectional area of the molten pool, which accelerates the cooling of the pool and thereby inhibits grain coarsening in the RHAZ. In other words, the heat dissipation control effect of the groove structure counteracts the grain-coarsening trend caused by the increased heat input. Consequently, there is no significant difference in the microstructure of the RHAZ between different layers.

### 3.3. Hardness Property of Multilayer Welded Joint

**Figure 12:**
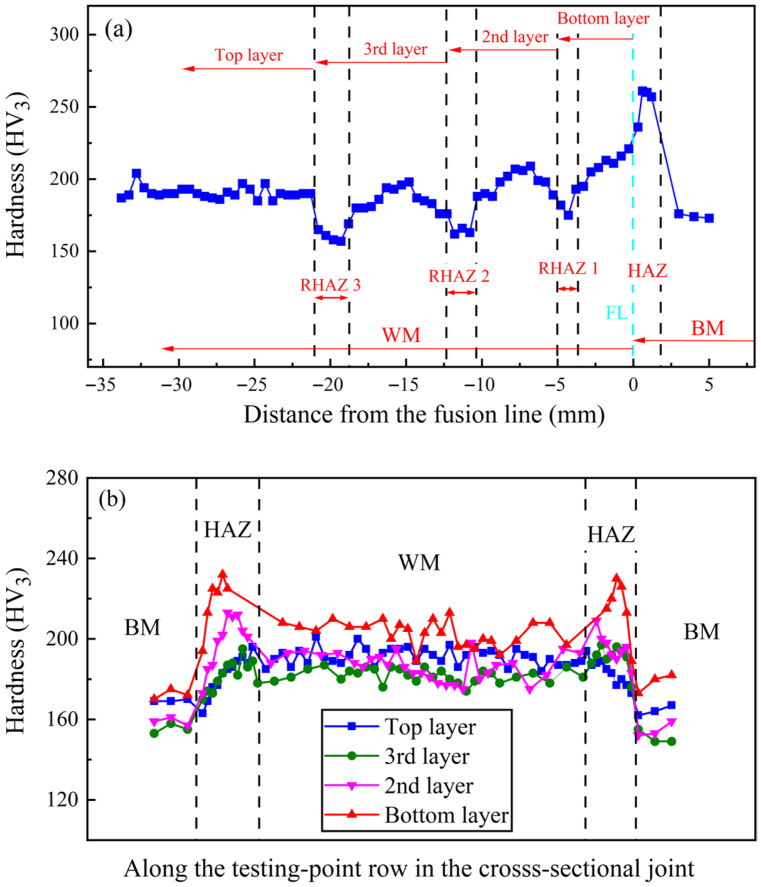
Hardness of multilayer welded joint: (**a**) vertical hardness distribution along the center of the welded joint, and (**b**) transverse hardness distributions across the weld layers.

Figure 12 presents the hardness distributions of the multilayer welded joint. As illustrated in Figure 12a, the hardness distribution along the vertical direction of the WM zone for each weld layer is relatively uniform with comparable values. This phenomenon can be attributed to the variable groove angle design, which enhances the cooling efficiency of the molten pool and thereby suppresses the weld grain-coarsening induced by the increased welding heat input, ultimately optimizing the hardness uniformity of each weld layer. In addition, three low-hardness regions were observed in the vertical hardness distribution (see Figure 12a), which correspond to the RHAZ formed between adjacent weld layers. Specifically, the top region of the preceding weld layer is reheated by the subsequent weld layer, leading to the softening of the microstructure in the RHAZ.

Figure 12b compares the hardness distributions along the transverse direction of each weld layer. The gradual decrease in the hardness of the WM zone from the bottom to the 3rd weld layer is primarily ascribed to the increased fraction of the low-hardness grain boundary ferrite (GBF) structure (see Figure 11b–d). This increase in GBF content raises the probability that the indentation of the hardness tester falls on the GBF region during transverse sampling along the weld, thereby reducing the measured hardness value. In contrast to the 2nd and 3rd weld layers, the hardness of the top weld layer is higher. This is because the top weld layer undergoes a faster cooling rate, which results in a finer grain structure in the WM zone (consistent with the microstructure characteristics described in Section 3.2.1) and thus improves the hardness.

Furthermore, the hardness variation trend in the sidewall HAZ is similar to that in the WM zone, which exhibits the highest value in the sidewall HAZ of the bottom layer, decreases gradually from the 2nd layer to the 3rd layer, and then increases slightly again in the top layer. This variation trend is consistent with the microstructure evolution of the sidewall CGHAZ elaborated in Section 3.2.2, confirming the close correlation between microstructure and hardness properties of the multilayer welded joint.

**Figure 13:**
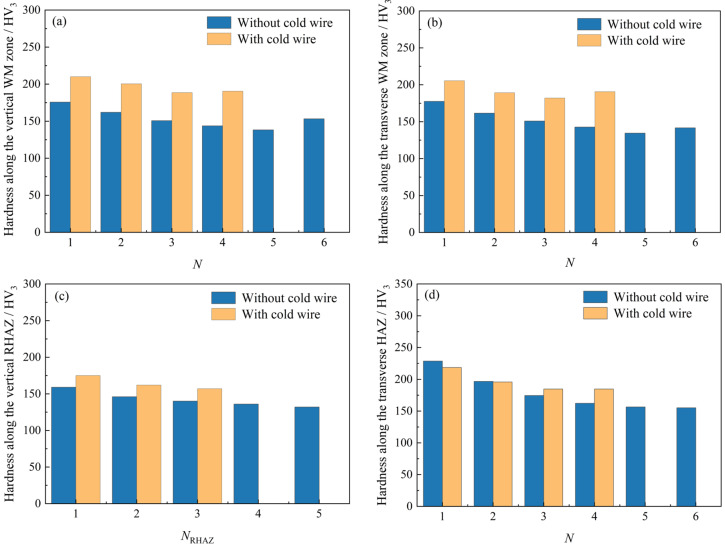
Regional hardness analysis of the multilayer welded joint with and without additional cold wire (corresponds to Figure 12): (**a**) hardness along the vertical WM zone, (**b**) hardness along the transverse WM zone, (**c**) hardness along the vertical RHAZ, and (**d**) hardness along the transverse HAZ; NRHAZ represents the RHAZ formed in the upper part of the *N*-th weld layer.

Figure 13 illustrates the regional hardness analysis of the multilayer welded joint with and without additional cold wires. Table 5 lists the standard deviation (SD) and coefficient of variation (CV) for hardness across each joint region. In WM zones, the hardness along both the vertical and transverse directions is higher (see Figure 13a,b) and more uniform (see Table 5) for the joint with pre-embedded cold wires than that without cold wire assistance. Correspondingly, the hardness uniformity of the WM zones along the vertical and transverse directions was increased by 48.3% and 62.1%, respectively. In RHAZs, the sample employing pre-embedded cold wires presents a higher hardness level (see Figure 13c) and a more homogeneous hardness distribution (see Table 5).

The increased hardness in the WM zone and RHAZ is attributed to the endothermic effect of cold wire melting, which, in conjunction with the arc-induced mechanical stirring effect, collectively regulates the heat distribution in the molten pool and refines the grain structure in the weld zone (including WM zone and RHAZ). Accordingly, the hardness uniformity of the RHAZ was increased by 33.3%. In addition, due to the pre-embedding cold wires, the hardness value in the sidewall HAZ of the bottom and 2nd layers is slightly lower; meanwhile, the hardness value in the 3rd layer and top layer is higher, as shown in Figure 13d. Consequently, the interlayer hardness difference was reduced (see Table 5), leading to a 54% improvement in the hardness uniformity of the sidewall HAZ. This improvement can be ascribed to the dual adjustment of the molten pool heat distribution by the adopted dual V-type groove structure and cold wire assistance. Specifically, the synergistic effect of heat absorption by cold wire melting and heat dissipation by the dual V-shaped groove makes the cooling rate of the molten pool more uniform, avoiding local overheating or undercooling, which in turn improves the microstructure and properties of the sidewall CGHAZ.

It follows that the variable-angle narrow-gap structure and additional cold wires not only optimize weld formation but also improve the hardness uniformity of the joint. Particularly, the hardness uniformity of the weakest HAZ of the joint is improved by 54%. This finding provides support for the subsequent comprehensive evaluation of joint performance.

## 4. Conclusions

A mathematical model of the welding energy density on the weld surface (*q_w_*) was established to predict the slag detachability. By adjusting the welding energy parameters and the pre-embedded cold wires, the *q_w_* was controlled below a threshold of 0.23 kJ/mm^2^ to ensure sound weld formation while yielding excellent deslagging. Particularly, the adoption of pre-embedded cold wires not only achieved a 33.0% reduction in the total number of weld layers but also shortened the welding time by 62.6%, both of which collectively contribute to a significant improvement in the efficiency of the multilayer welding process.

The slag adhesion mechanism of the NG-SAW welded joint was clarified. High welding heat input prolongs the high-temperature dwell time of the weld metal, thereby enhancing elemental diffusion at the weld–slag interface and promoting the formation of a continuous, thick interlayer composed of (Fe,Mn)O and MgO-Al_2_O_3_-CaO phases. This interlayer strengthens the chemical bonding between slag and weld, hindering the separation of slag from the weld and making slag removal difficult.

The microstructural evolution and hardness distribution characteristics of the multilayer welded joint were systematically revealed. The weld metal (WM) zone is primarily composed of acicular ferrite and grain boundary ferrite. The adoption of a variable-angle design enlarges the groove volume and, combined with the heat-absorbing effect of additional wires, accelerates the cooling rate of the molten pool, thus leading to obvious grain refinement in both the WM zone and reheat-affected zone. Meanwhile, the tempering effect exerted by the subsequent weld layer on the sidewall heat-affected zone (HAZ) of the preceding layer is weakened, which promotes the gradual transformation from hard-brittle lath martensite in the sidewall coarse-grained heat-affected zone (CGHAZ) of the bottom layer to tougher tempered martensite/bainite in the corresponding regions of the upper layers. Consequently, the hardness uniformity within the HAZ—the critical weak region of the joint—is significantly improved by 54%, contributing to favorable microstructural and mechanical property optimization of the welded joint, while also enhancing efficiency of the overall welding process.

## Figures and Tables

**Table 1:** Chemical compositions of the base metal and the filler material (wt%).

	C	Mn	Si	Cr	Ni	Al	Ti	Nb	Cu	V	S	P
EH36	0.07	1.49	0.13	0.013	0.34	0.004	0.018	0.014	0.05	0.01	0.003	0.011
CHW-S3	0.074	1.80	0.04	0.03	0.02	-	-	0.03	0.01	-	0.003	0.013

**Table 2:** Welding parameters for single-layer submerged arc welding process.

Factor	Additional Cold Wire Layer (*n*)	Welding Current (*I_a_*, A)	Arc Voltage (*U_a_*, V)	Welding Speed (*V_w_*, mm/min)	Arc Swing Frequency (*f*, Hz)	Sidewall Staying Time (*t_s_*, ms)	Reserved Gap (*g*, mm)	Arc Swing Angle (*α*, °)
Additional cold wire	0, 1, 2, 3	658	29.6	400	4.0	50	2.0	25
Welding heat input	3	607, 658, 708	29.6	350, 400, 450	4.0	50	2.0	25

**Table 3:** Welding parameters for multilayer submerged arc welding process.

Weld Layer *N*	Additional Wire Layer (*n*)/Total Wire Number (*n_p_*)	Welding Current (*I_a_*, A)	Arc Voltage (*U_a_*, V)	Welding Speed (*V_w_*, mm/min)	Arc Swing Frequency (*f*, Hz)	Sidewall Staying Time (*t_s_*, ms)	Reserved Gap (*g*, mm)	Arc Swing Angle (*α*, °)
1	3/16	658	29.6	400	4.0	50	2.0	25
2	3/22	750	31.8					42
3	3/25	810	32.7					52
4	3/28	870	34.6					71

**Table 4:** Slag detachability under different welding heat inputs.

Welding Heat Input (kJ/cm)	Reaction Interlayer Characteristics	Resultant Force (*F_R_*)	Slag Detachability
26.1	Ultra-thin or non	≈0	Easy
29.3	Thin	<0	Moderate
33.5	Thick	<<0	Difficult

**Table 5:** Comparison of standard deviation (SD) and coefficient of variation (CV) for hardness across each joint region.

	WM_0 ver._	WM_cw ver._	WM_0 tra._	WM_cw tra._	RHAZ_0_	RHAZ_cw_	HAZ_0_	HAZ_cw_
SD	12.3	8.6	14.4	7.0	9.4	7.6	26.3	13.9
CV	8.0%	4.1%	9.5%	3.6%	6.6%	4.4%	14.7%	6.8%

Note: subscript _0_—the joint without cold wire addition, subscript _cw_—the joint with cold wire assistance, subscript _ver._—the hardness along the vertical direction of the WM zone, and subscript _tra._—the hardness along the transverse direction of the WM zone.

## Data Availability

The original contributions presented in this study are included in the article. Further inquiries can be directed to the corresponding authors.
